# Longitudinal analysis of dental healthcare costs among adolescents living in Amsterdam

**DOI:** 10.2340/aos.v85.46952

**Published:** 2026-10-06

**Authors:** Tessa S. van Ligten, Catherine M.C. Volgenant, Egija Zaura, Denise Duijster

**Affiliations:** a Department of Cariology, Academic Centre for Dentistry Amsterdam (ACTA), University of Amsterdam and Vrije Universiteit, Amsterdam, The Netherlands; b Department of Preventive Dentistry, Academic Centre for Dentistry Amsterdam (ACTA), University of Amsterdam and Vrije Universiteit, Amsterdam, The Netherlands; c Department of Oral Public Health, Academic Centre for Dentistry Amsterdam (ACTA), University of Amsterdam and Vrije Universiteit, Amsterdam, The Netherlands

**Keywords:** Regular dental visits, dental healthcare, epidemiology, oral healthcare consumption, public dental health, dental care for children, dental health services, health care costs

## Abstract

**Objectives:**

The aim was to analyze patterns of oral healthcare consumption from 2009 to 2016 of adolescents living in Amsterdam. It was assessed whether visiting a dentist earlier in childhood and regular dental attendance between ages 10 and 16 were associated with oral healthcare consumption at age 17. Dental costs (in euros) were used as a proxy for oral healthcare consumption since data on dental performance codes, number of visits, or clinical data were not available in this study.

**Methods:**

The study population consisted of all 12-year-old adolescents living in Amsterdam in 2012. Retrospective, longitudinal data on dental costs and sociodemographics were obtained via Statistics Netherlands. Explanatory variables were whether dental costs were incurred at age 9 (in 2009; yes/no and amount) and whether consecutive dental costs were incurred from the age of 10 to 16 (between 2010 and 2015; yes/no). The outcome was dental costs at age 17 (in 2016; yes/no and amount).

**Results:**

Dental costs at age 17 were available for 6,871 children. Dental costs at age 9 and consecutive dental costs were significantly associated with having dental costs at age 17. The amount of dental costs at age 9 was significantly and positively associated with higher dental costs at age 17. No association was found between consecutive dental costs during childhood and the amount of dental costs at age 17.

**Conclusions:**

A large volume of oral healthcare consumption earlier in childhood was associated with high dental costs for 17-year-olds. Adolescents who regularly visited a dentist during childhood continued to do so at age 17. However, regular dental attendance was not associated with lower dental costs at age 17.

## Introduction

Dental costs vary considerably between countries worldwide, with an unequal spending pattern that favors high-income countries: 99 low- and middle-income countries spend very little on oral healthcare (on average 1–10 US dollars per person annually), while an average of more than 300 US dollars per person is spent per year in 12 countries with the highest dental expenditures. In high-income countries, a total amount of 340 billion US dollars is incurred for oral healthcare, which is 88% of the total of dental costs globally, but only covers 22.5% of the global population [1]. Within Europe, it is common to visit a dentist regularly. In the Netherlands, the average is 3.1 dental visits per person in 2021 (in Europe range: 0.6 to 1.7 dental visits annually [2]). In the Netherlands, the dental costs were 190 US dollars per person (in Europe range: 75 US dollars to 432 US dollars across countries in 2019 [3]). Yet, for persons who visit a dentist in the Netherlands, the average dental expenditure per person annually is between 240 and 272 US dollars [4].

Regular dental check-ups (at least once per year) have been advised to monitor oral health and detect potential risks early. If needed, preventive care and restorative treatment can be provided in a timely manner [5, 6]. Previous research has shown that routine dental attendance is associated with better oral health, and opportunistic, non-regular dental attendance is associated with more dental caries in young adults compared to regular dental attendance [7]. Changing dental visiting behavior from opportunistic, non-regular dental attendance toward regular dental attendance is associated with improved oral health, especially for patients with a low socioeconomic position (SEP) [8, 9]. However, a recent scoping review by Amarasena et. al. [10] stated that there is very little evidence that regular dental attendance is associated with oral diseases such as dental caries and periodontitis later in adulthood and suggested that the frequency of dental visits should be individually tailored and based on a risk assessment for each patient.

The frequency of dental visits changes throughout life, based on age. In a study by Thomson et. al. from New Zealand, the percentage of people who regularly attend a dentist dropped significantly from 82% in 15-year-olds to 28% in 32-year-olds [7]. According to a Dutch national survey by Statistics Netherlands, on average, four out of five Dutch adults visit a dentist every year [11, 12]. When split into age groups, only 43.3% of the 2- and 3-year-old toddlers visited a dentist in 2021, while the majority of 4- to 11-year-old children and 12- to 17-year-old adolescents regularly attended a dentist in that year (85%, 84% respectively) [11]. Yet, the proportion of children and adolescents with an annual dental check-up in Amsterdam, the capital of the Netherlands, was slightly lower than the national average (26% vs. 39%; 2 to 3 years; 80% vs. 82%; 4 to 11 years, and 77% vs. 81%; 12 to 17 years) [13]. In the Netherlands, oral healthcare is mostly privately funded for adults, either through out-of-pocket payments or through additional insurance. For children and adolescents until the age of 18, oral healthcare is covered by the mandatory basic medical insurance until 18 years. Therefore, there are no direct financial barriers to visiting a dentist as a child or adolescent [14]. However, practical, social, cultural, organizational, or informational barriers may remain.

In the Netherlands, routine dental check-ups remain common practice, which provides an opportunity to reach children during critical stages of growth and development for preventive interventions, provided these visits are used effectively [15]. Our previous study focused on younger children: during early childhood, if children go to a dentist for a first visit before the age of four, and thereafter go regularly, the oral healthcare consumption throughout childhood is similar to children without early or regular dental visits [16], which is in disagreement with the rationale that prevention leads to a lower treatment need [17]. This study extends the analysis into adolescence and the period just before transition to adult oral healthcare. Little is known about how patterns of dental attendance affect oral health specifically in adolescence. Despite full coverage for oral healthcare for youth, previous research showed that among adolescents, still a large proportion has significant caries experience, and that burden falls disproportionally onto children from low SEP families [18]. That raises the question why there is such variation in patterns of oral healthcare consumption among adolescents, and whether the variation leads to different oral health outcomes, and if so, which factors may play a role.

The aim of this study was to analyze patterns of oral healthcare consumption from 2009 to 2016 of adolescents living in Amsterdam. It was assessed whether visiting a dentist earlier in childhood and regular dental attendance between ages 10 and 16 were associated with oral healthcare consumption at age 17. Dental costs (in euros) were used as a proxy for oral healthcare consumption since data on dental performance codes, number of visits, or clinical data were not available in this study. Incurring dental costs was a proxy indicator for visiting the dentist, and no dental costs indicated no dental visit. The amount of dental costs was used as a proxy for the volume of oral healthcare provided, with higher costs likely indicating a higher volume of preventive care and restorative treatment.

The following research questions were studied:

- Was the amount of dental costs at the age of 9 associated with having a dental visit at age 17?
- For those who did have a dental visit at age 9, was the amount of dental costs at age 9 associated with the amount of dental costs at age 17?
- Was regular dental attendance between the ages of 10 and 16 associated with having a dental visit at age 17, and if so, with the amount of dental costs at age 17?

## Methods

### Population

This study was a collaboration between the Academic Centre for Dentistry Amsterdam (ACTA) and the City of Amsterdam, the Netherlands. Data were obtained from a large dataset from Statistics Netherlands with all children living in Amsterdam from one up to 17 years of age. This dataset comprised only the data collected between 2009 and 2016. Therefore, for children aged 12 in 2012, the earliest available data point was at age 9 in 2009, which constrained our analysis of earlier childhood dental visits since data prior to the age of nine were not available. Oral healthcare consumption and dental service use during early childhood in relation to preventive patterns was analyzed and published separately. The study population of our previous publication contained all 4-year-old children registered in Amsterdam on January 1, 2012, and those children were followed from the age of 1 year up until the age of 8 years [16]. The population of this study consisted of all 12-year-old adolescents who were registered in Amsterdam, the Netherlands, on January 1, 2012. From this sample, data on dental costs and sociodemographic characteristics were available from Statistics Netherlands from January 1, 2009, to December 31, 2017, hence resulting in a retrospective, longitudinal dataset with a follow-up of those adolescents from 9 to 17 years of age. Adolescents who moved out of Amsterdam during the follow-up period were excluded from the study. The aims and design of the study are schematically presented in Figure 1. The study protocol was approved by the internal ethics committee of ACTA (protocol number 2023-98097).

**Figure 1:**
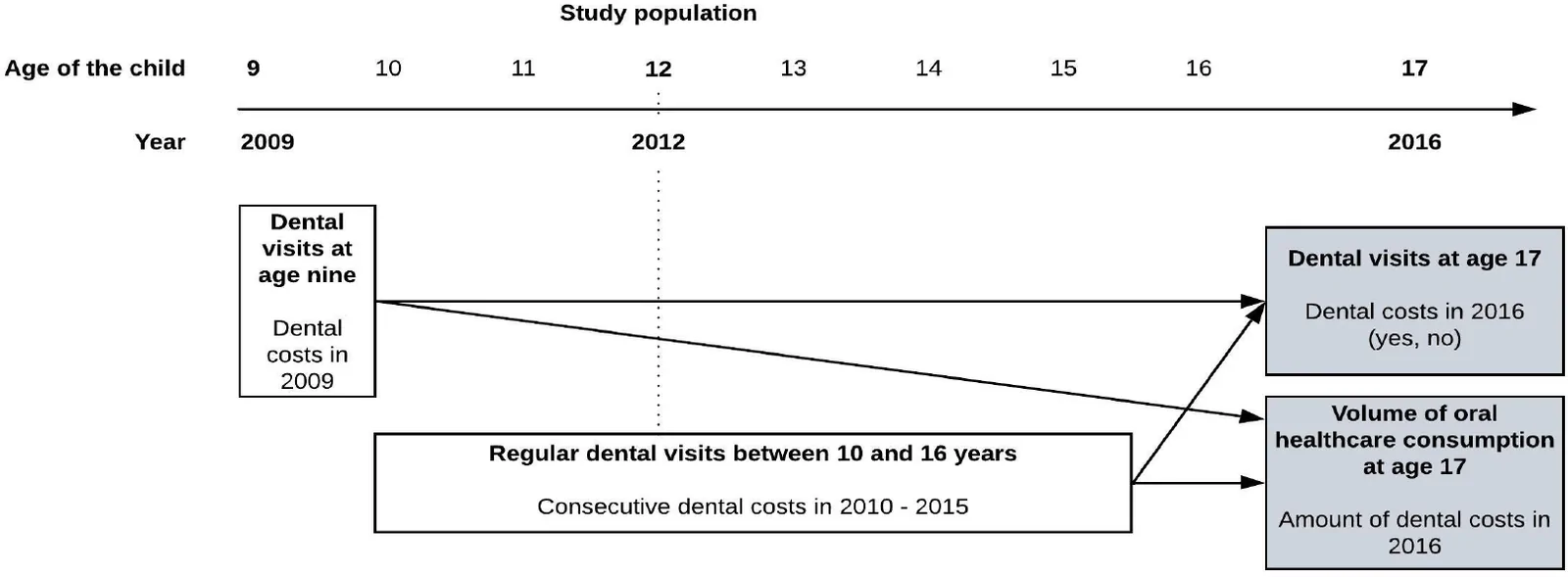
Schematic illustration of the aims and design of the study.

### Research data

Oral healthcare consumption of a child is the total volume of oral healthcare provided from 2009 to 2016, during which dental consultations and both preventive care and restorative treatments were provided. Yet, in this study, dental costs (in euros) were used as a proxy for oral healthcare consumption, since data on dental performance codes, number of visits, and clinical data were not available through Statistics Netherlands. Dental costs were registered as the amount per year per adolescent, incurred via the basic medical insurance. Basic medical insurance is compulsory for all Dutch citizens and covers oral healthcare up to the age of 17 years. The source of dental costs was a business intelligence center that holds all healthcare reimbursement data of healthcare insurance companies in the Netherlands (Vektis). Dental costs did not include orthodontic treatment because it is not covered under the basic medical insurance. Data on sociodemographic characteristics were obtained from the Dutch Personal Records Database (Basisregistratie personen; BRP), the Education Executive Agency of the Ministry of Education, Culture and Science (Dienst Uitvoering Onderwijs; DUO), and the Tax Authorities. Each participant’s citizen service number was used to link data from various sources by Statistics Netherlands, and thereafter the citizen service number was replaced by a unique identification code in the research file. Children and adolescents do not have their own basic medical insurance. They are covered under one of their parent’s mandatory insurance policies. In the rare occasion that both parents were not insured, the adolescents were excluded from this study.

#### Outcomes

The outcome in this study was dental costs of 17-year-old adolescents, incurred in 2016. Data on dental costs from 2016 were used instead of 2017, since a part of the sample turned 18 in 2017, which meant that part of that year’s dental costs were no longer covered under the basic medical insurance and that data were therefore not present in the dataset of that year. Analysis indeed showed that a substantially smaller percentage of adolescents incurred dental costs in 2017 than in 2016 (63% vs. 76%). For those with dental costs, the distribution of dental costs was similar for both years. First, it was analyzed whether dental costs were incurred in 2016 (dichotomous; yes or no) and second, for the adolescents who did visit a dentist, the amount of dental costs incurred in 2016 (continuous).

#### Independent variables

The explanatory variables were oral healthcare consumption at age 9 (earliest year in childhood with available data) and regular dental attendance from 10 to 16 years of age (continuous years from age 10 up to 16). Whether a dental visit took place at age 9 was defined as dental costs incurred in 2009 (yes/no), and if dental costs were incurred, the amount of dental costs was categorized (no dental costs, > 0 to < 100 euros, ≥ 100 to < 300 euros, ≥ 300 euros, and unknown/other). Also, the amount of dental costs at age 9 was analyzed (continuous variable). The categories were pragmatically based on the Dutch performance codes for dental costs in 2016: periodic examination (20.08 euros), preventive care per 5 min (11.85 euros), a single-surface composite filling (42.28 euros) and multiple-surface composite filling (84.56 euros). Children with dental costs only for consultation and preventive care, most likely did not exceed 100 euros annually. If additional dental treatment was needed, dental costs would be over 100 euros and for children with an extensive treatment need with multiple restorations required, dental costs would be more than 300 euros [19]. For regular dental attendance, consecutive dental costs from 2010 to 2015 were used as a proxy measure and were defined as dental costs incurred in each year, or with a maximum of 1 year missing, versus ≥2 years with no dental costs. In the Netherlands, the guideline for oral healthcare for children advises visiting a dentist for a check-up at least once a year [6]. To include children who had an interval between dental costs of just over 1 year, 1 year missing was allowed and therefore the cut-off was defined as dental costs incurred in each year or with a maximum of 1 year missing versus ≥ 2 years with no dental costs.

#### Sociodemographic characteristics

Based on the literature, the following sociodemographic characteristics were selected from the dataset to include in the analyses: sex, migration background, level of education, type of household, household income, and parental educational level. These variables were obtained from the BRP, DUO, and Tax Authorities. Migration background was based on the parental country of birth, categorized as: no migration background, Western migration background, and non-Western migration background (BRP) [20]. The educational level at age 17 was based on the Dutch school system for adolescents: lower secondary education, higher secondary education, lower vocational education and higher vocational/university education, and unknown/other. Because of the small number of adolescents with primary school or the first 3 years of higher secondary education as the highest level, those were included in the unknown/other category (DUO) [21]. The type of household was categorized as a two-parent family, a single-parent household, and unknown/other (BRP). Household income was divided into quintiles: 0–20%, 21–40%, 41–60%, 61–80%, 81–100%, based on the national household income and an unknown/other category (Tax Authorities). Parental educational level was split into low, medium, high, and unknown/other [22].

### Statistical analysis

Descriptive data were used to present dental costs from 2009 to 2017 and sociodemographic characteristics. First, univariable and multivariable logistic regression analyses were performed to assess associations of dental costs at age 9 (categorical) and consecutive dental costs between 2010–2015 with incurred dental costs at age 17 (yes/no). Second, for the group that incurred dental costs at age 17, univariable and multivariable linear regression analyses were performed to assess if dental costs at age 9 (continuous) and consecutive dental costs for 2010–2015 were associated with the amount of dental costs at age 17 (continuous). Both logistic and linear multivariable analyses were adjusted for the sociodemographic characteristics: sex, migration background, child’s education, type of household, household income, and parental educational level. The variables were selected based on the literature and were entered in a single step in the analyses (Enter Method).

The normal distribution did not apply to our data (eyeballing). To check the robustness of the data, the analyses of the original data and data transformed with a natural logarithm were compared. The results did not differ significantly. Hence, the decision was made to analyze the original, non-transformed data since those results are presented in mean difference in euros and are easier to interpret than the geometrical mean differences from log-transformed data. The statistical analyses were conducted in IBM SPSS Statistics v25 [23].

#### Missing data and data integrity

Missing data on the outcome (dental costs at age 17; incurred in 2016) were excluded from analyses (*n* = 353 [4.65%]). To minimize potential bias from extreme outliers, a small proportion of adolescents with very high dental costs (≥ 1,500 euros; mean dental costs in 2016 were 255.63 euros) were excluded from the data (*n* = 89 [1.17%]). These cases typically reflect complex clinical situations beyond the scope of routine (preventive) care, which was the primary focus of this study. Missing data in the independent variables were not excluded, but were categorized as unknown, and combined with the category ‘other’ and analyzed as such. The category unknown/other was < 6% for the independent variables, except for parental educational level (35%) due to limited recognition of foreign diplomas in the Netherlands (Table 1).

**Table 1:** Descriptive data of 12-year-old children in 2012, overall and categorized by dental costs at age 9 and consecutive dental costs.

	*N* (% of variable)	Dental costs at age 9			Consecutive dental costs between ages 10 and 16	
		No (%)	Yes (%)	*N* (% of variable)	No (≥ 2 years no dental costs incurred) (%)	Yes (%)
**Total**	**7,151**	**1,082**	**6,069**	**6,805**	**2,034**	**4,771**
**Child variables**						
**Sex**						
Boy	3,595 (50.3)	565 (52.2)	3,030 (49.9)	3,407 (50.1)	1,081 (53.1)	2,326 (48.8)
Girl	3,556 (49.7)	517 (47.8)	3,039 (50.1)	3,398 (49.9)	953 (46.9)	2,445 (51.2)
**Migration background**						
No migration background	2,480 (34.7)	388 (35.9)	2,092 (34.5)	2,442 (35.9)	645 (31.7)	1,797 (37.7)
Western migration background	663 (9.3)	127 (11.7)	536 (8.8)	592 (8.7)	184 (9.0)	408 (8.6)
Non-Western migration background	4,008 (56.0)	567 (52.4)	3,441 (56.7)	3,771 (55.4)	1,205 (59.2)	2,566 (53.8)
**Education of the child**						
Lower secondary education	846 (11.8)	142 (13.1)	704 (11.6)	823 (12.1)	308 (15.1)	515 (10.8)
Higher secondary education	2,871 (40.1)	410 (37.9)	2,461 (40.6)	2,830 (41.6)	720 (35.4)	2,110 (44.2)
Lower vocational education	2,738 (38.3)	343 (31.7)	2,395 (39.5)	2,718 (39.9)	884 (43.5)	1,834 (38.4)
Higher vocational or university education	288 (4.0)	35 (3.2)	253 (4.2)	287 (4.2)	63 (3.1)	224 (4.7)
Unknown/other	408 (5.7)	152 (14.0)	256 (4.2)	147 (2.2)	59 (2.9)	88 (1.8)
**Type of household**						
Two-parent family	4,715 (65.9)	663 (61.3)	4,052 (66.8)	4,496 (66.1)	1,191 (58.6)	3,305 (69.3)
Single-parent	2,360 (33.0)	401 (37.1)	1,959 (32.3)	2,237 (32.9)	807 (39.7)	1,430 (30.0)
Unknown/other	76 (1.1)	18 (1.7)	58 (1.00)	72 (1.1)	36 (1.8)	36 (0.8)
**Parent variables**						
**Household income**						
Quintile (0–20%)	2,886 (40.4)	379 (35.0)	2,507 (41.3)	2,742 (40.3)	888 (43.7)	1,854 (38.9)
Quintile (21–40%)	1,282 (17.9)	180 (16.6)	1,102 (18.2)	1,268 (18.6)	390 (19.2)	878 (18.4)
Quintile (41–60%)	1,024 (14.3)	149 (13.8)	875 (14.4)	1,011 (14.9)	281 (13.8)	730 (15.3)
Quintile (61–80%)	755 (10.6)	105 (9.7)	650 (10.7)	745 (10.9)	178 (8.8)	567 (11.9)
Quintile (81–100%)	965 (13.5)	173 (16.0)	792 (13.0)	936 (13.8)	247 (12.1)	689 (14.4)
Unknown/other	239 (3.3)	96 (8.9)	143 (2.4)	103 (1.5)	50 (2.5)	53 (1.1)
**Educational level of the parent**						
Low	1,622 (22.7)	214 (19.8)	1,408 (23.2)	1,537 (22.6)	519 (25.5)	1,018 (21.3)
Medium	1,408 (19.7)	210 (19.4)	1,198 (19.7)	1,362 (20.0)	429 (21.1)	933 (19.6)
High	1,561 (21.8)	252 (23.3)	1,309 (21.6)	1,507 (22.1)	393 (19.3)	1,114 (23.3)
Unknown/other*	2,560 (35.8)	406 (37.5)	2,154 (35.5)	2,399 (35.3)	693 (34.1)	1,706 (35.8)

* Educational level of the parent had a high percentage of missing data (35%) due to limited recognition of foreign diplomas in the Netherlands.

## Results

The study population consisted of 6,871 adolescents, after exclusion of those with missing data on dental costs at age 17, incurred in 2016, and those with very high dental costs (≥ 1,500 euros). Sociodemographic characteristics of the study sample and the distribution of dental costs at age 9 and consecutive dental costs between age 10 and 16 are shown in Table 1. The percentage of adolescents incurring dental costs slightly decreased from age 9 to 17 (84.9 to 76.0%; Figure 2a). For those with dental costs, the mean amount (in euros) steadily increased over the years (2009: 176.86; 2016: 255.63; Figure 2b). Data on dental costs at age 9 were available for 7,151 persons and consecutive dental costs for 6,805 adolescents. Boys and girls were evenly distributed; more than half of the adolescents had a non-Western migration background, and the majority followed either higher secondary education or lower vocational education. Two-thirds lived in a two-parent family, and a relatively large proportion grew up in a family with a household income in the lowest quintile. Parental education was fairly evenly distributed across the levels: low, medium, and high. For the 6,810 adolescents who incurred dental costs in 2009, 2012, and 2016, the distribution of dental costs at age 9, 12, and 17 is shown in Figure 3.

**Figure 2:**
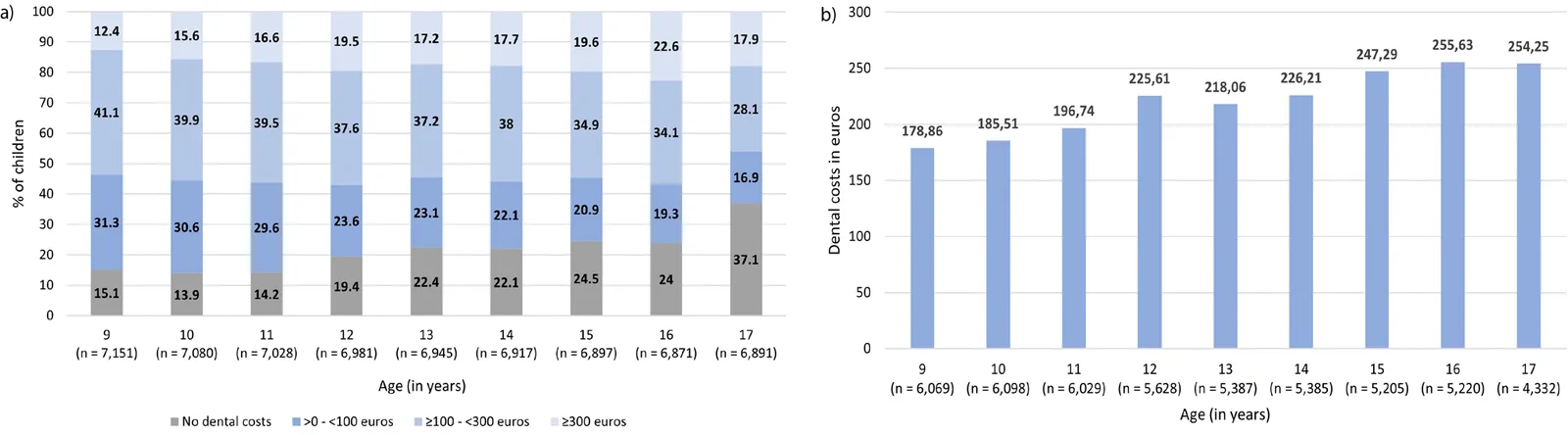
Dental costs incurred in adolescents: (a) distribution of the dental costs per age (in years) divided into: no dental costs, > 0 – < 100 euros, ≥ 100 – < 300 euros, and ≥ 300 euros. (b) mean dental costs in euros per age (in years), in the group of children that incurred dental costs.

**Figure 3:**
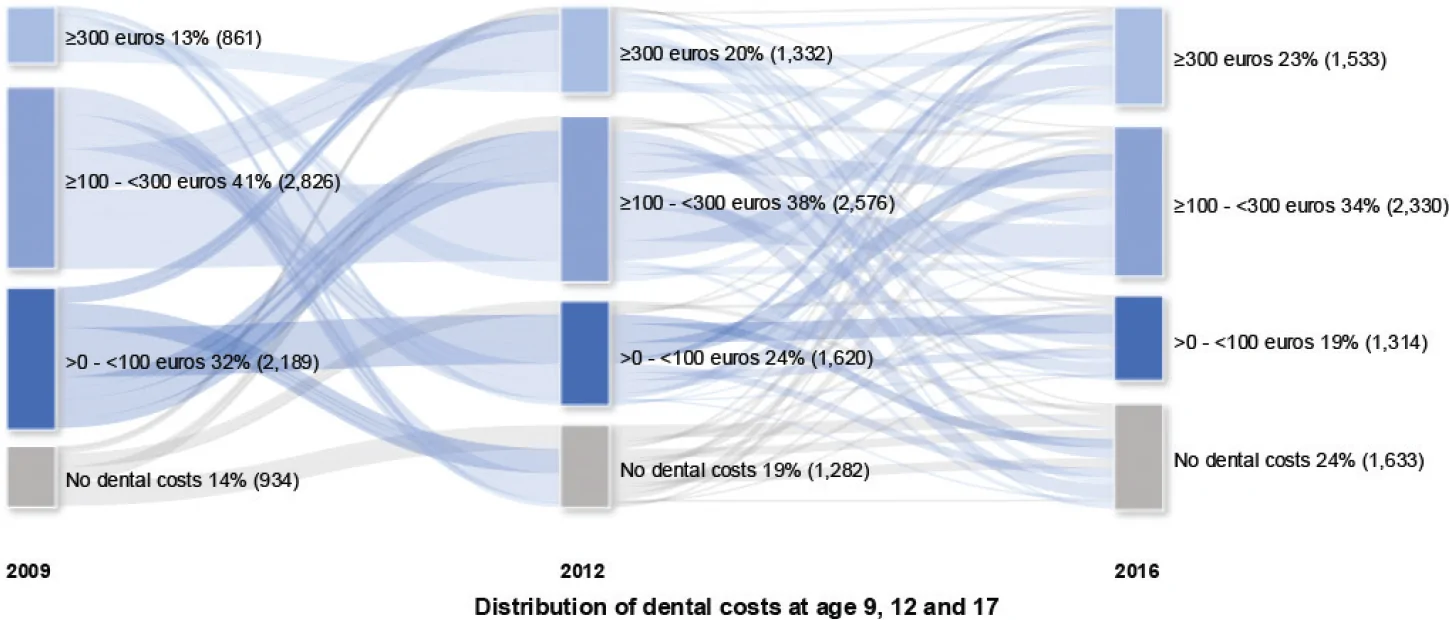
Distribution of dental costs at ages 9, 12, and 17.

### Dental costs at age 9 and consecutive dental costs and associations with (no) dental costs at age 17

Dental costs at age 9 were significantly associated with incurring dental costs at age 17 (vs. no dental costs). Results showed that adolescents with dental costs at age 9 (> 0 to < 100 euros, ≥ 100 to < 300 euros, and ≥ 300 euros) had higher odds of incurring dental costs at age 17 than those who did not incur dental costs at age 9 (adjusted odds ratio [aOR] range 2.01 [1.70–2.37] – 3.04 [2.44–3.80]). Incurring consecutive dental costs between ages 10 and 16 was significantly associated with incurring dental costs at age 17 (aOR 5.24 [4.64–5.92]). For both analyses, adolescents with a non-Western migration background or living with a single parent had a lower likelihood of incurring dental costs at age 17. When attending higher vocational or university education, more adolescents incurred dental costs at age 17. Additionally, regarding the analysis of dental costs at age 9, attending higher secondary education and a high parental educational level were associated with dental costs at age 17, compared to no dental costs (Tables 2 and 3).

**Table 2:** Dental costs (vs. no dental costs) in 2016 and the amount of dental costs in 2016 and associations with dental costs at age 9, crude analyses and analyses adjusted for sociodemographic characteristics.

	Dental costs vs. no dental costs in 2016			Amount of dental costs in 2016		
	Crude (Univariable logistic regression)	Adjusted (Multivariable logistic regression)		Crude (Univariable linear regression)	Adjusted (Multivariable linear regression)	
	OR	aOR	*p*	B	B	*p*
**Dental costs at age 9**						
No	reference	reference		–	–	–
> 0 to < 100 euros	2.13	2.01 (1.70–2.37)	< 0.001	–	–	–
≥ 100 to < 300 euros	2.21	2.31 (1.96–2.71)	< 0.001	–	–	–
≥ 300 euros	2.69	3.04 (2.44–3.80)	< 0.001	–	–	–
Unknown/other	1.66	1.87 (0.71–4.93)	0.207	–	–	–
**Dental costs at age 9**						
Dental costs continuous	–	–	–	0.28	0.19 (0.15–0.23)	< 0.001
**Child variables**						
**Sex**						
Boy	reference	reference		reference	reference	
Girl	1.13	1.10 (0.98–1.23)	0.095	−4.20	−4.22 (−16.57 to 8.12)	0.502
**Migration background**						
No migration background	reference	reference		reference	reference	
Western migration background	0.89	0.89 (0.71–1.11)	0.291	28.80	23.27 (0.05–46.49)	0.050
Non-Western migration background	0.70	0.74 (0.64–0.85)	<0.001	103.87	51.96 (36.56–67.36)	< 0.001
**Education of the child**						
Lower secondary education	reference	reference		reference	reference	
Higher secondary education	1.61	1.32 (1.10–1.60)	0.004	−111.09	−66.42 (−88.27 to −44.57)	< 0.001
Lower vocational education	1.14	1.08 (0.91–1.29)	0.400	−31.98	−35.86 (−56.87 to −14.86)	0.001
Higher vocational or university education	2.13	1.84 (1.29–2.63)	0.001	−105.78	−78.19 (−112.49 to −43.90)	< 0.001
Unknown/other	0.85	0.83 (0.54–1.26)	0.383	−110.55	−74.46 (−129.94 to −18.97)	0.009
**Type of household**						
Two-parent family	reference	reference		reference	reference	
Single-parent	0.66	0.74 (0.65–0.84)	<0.001	6.39	−9.42 (−23.61 to 4.77)	0.193
Unknown/other	0.67	0.92 (0.52–1.62)	0.763	31.63	−26.34 (−101.22 to 48.54)	0.491
**Parent variables**						
**Household income**						
Quintile (0–20%)	reference	reference		reference	reference	
Quintile (21–40%)	1.10	1.04 (0.89–1.22)	0.609	−31.74	−9.45 (−27.03 to 8.12)	0.292
Quintile (41–60%)	1.11	0.96 (0.80–1.14)	0.631	−56.04	−16.01 (−35.68 to 3.66)	0.111
Quintile (61–80%)	1.50	1.12 (0.90–1.40)	0.303	−90.93	−26.86 (−49.51 to −4.21)	0.020
Quintile (81–100%)	1.51	1.05 (0.84–1.31)	0.657	−118.38	−32.91 (−55.91 to −9.91)	0.005
Unknown/other	0.75	0.91 (0.60–1.38)	0.664	34.95	52.06 (−6.81 to 110.94)	0.083
**Educational level of the parent**						
Low	reference	reference		reference	reference	
Medium	1.18	1.09 (0.92–1.30)	0.317	−77.57	−35.83 (−55.66 to −16.00)	< 0.001
High	1.62	1.23 (1.01–1.51)	0.043	−131.40	−49.21 (−71.00 to −27.43)	< 0.001
Unknown/other	1.28	1.14 (0.98–1.33)	0.095	−45.31	−14.26 (−31.71 to 3.19)	0.109

OR: odds ratio; aOR: adjusted odds ratio.

**Table 3:** Dental costs (vs. no dental costs) in 2016 and the amount of dental costs in 2016 and associations with consecutive dental costs, crude analyses and analyses adjusted for sociodemographic characteristics.

	Dental costs vs. no dental costs in 2016			Amount of dental costs in 2016		
	Crude (Univariable logistic regression)	Adjusted (Multivariable logistic regression)		Crude (Univariable linear regression)	Adjusted (Multivariable linear regression)	
	OR	aOR	*p*	B	B	*p*
**Consecutive dental costs incurred (in years)**						
≥ 2 years no dental costs incurred	reference	reference		reference	reference	
Consecutive dental costs incurred	5.48	5.24 (4.64–5.92)	< 0.001	−20.52	−13.29 (−28.00 to 1.42)	0.077
**Child variables**						
**Sex**						
Boy	reference	reference		reference	reference	
Girl	1.13	1.06 (0.94–1.20)	0.333	−4.20	−0.39 (−12.30 to 11.52)	0.948
**Migration background**						
No migration background	reference	reference		reference	reference	
Western migration background	0.89	1.03 (0.81–1.31)	0.790	28.80	23.48 (1.33–45.63)	0.038
Non-Western migration background	0.70	0.81 (0.70–0.94)	0.006	103.87	61.21 (46.59–75.84)	< 0.001
**Education of the child**						
Lower secondary education	reference	reference		reference	reference	
Higher secondary education	1.61	1.21 (0.99–1.48)	0.068	−111.09	−64.27 (−85.18 to −43.36)	< 0.001
Lower vocational education	1.14	1.09 (0.90–1.31)	0.395	−31.98	−30.79 (−50.94 to −10.64)	0.003
Higher vocational or university education	2.13	1.59 (1.10–2.31)	0.014	−105.78	−79.73 (−112.79 to −46.67)	< 0.001
Unknown/other	0.85	0.88 (0.55–1.40)	0.576	−110.55	−87.41 (−139.40 to −35.42)	0.001
**Type of household**						
Two-parent family	reference	reference		reference	reference	
Single-parent	0.66	0.79 (0.69–0.90)	< 0.001	6.39	−14.08 (−27.66 to −0.50)	0.042
Unknown/other	0.67	1.04 (0.56–1.93)	0.909	31.63	−24.78 (−93.30 to 43.75)	0.479
**Parent variables**						
**Household income**						
Quintile (0–20%)	reference	reference		reference	reference	
Quintile (21–40%)	1.10	1.00 (0.84–1.18)	0.980	−31.74	−15.30 (−32.25 to 1.64)	0.077
Quintile (41–60%)	1.11	0.87 (0.72–1.05)	0.152	−56.04	−23.77 (−42.66 to −4.87)	0.014
Quintile (61–80%)	1.50	1.00 (0.79–1.27)	0.988	−90.93	−35.76 (−57.51 to −14.01)	0.001
Quintile (81–100%)	1.51	1.02 (0.81–1.29)	0.855	−118.38	−44.58 (−66.39 to −22.78)	< 0.001
Unknown/other	0.75	1.11 (0.65–1.87)	0.707	34.95	30.27 (−27.66 to 88.21)	0.306
**Educational level of the parent**						
Low	reference	reference		reference	reference	
Medium	1.18	1.07 (0.89–1.29)	0.470	−77.57	−42.02 (−61.26 to −22.78)	< 0.001
High	1.62	1.18 (0.95–1.47)	0.128	−131.40	−54.15 (−75.15 to −33.16)	< 0.001
Unknown/other	1.28	1.09 (0.92–1.28)	0.321	−45.31	−17.12 (−34.05 to −0.19)	0.048

OR: odds ratio; aOR: adjusted odds ratio.

### Dental costs at age 9 and consecutive dental costs and associations with the amount of dental costs at age 17

A higher amount of incurred dental costs at age 9 was significantly associated with incurring higher dental costs at age 17 (mean difference 0.19 euros [0.15–0.23]). Adolescents with consecutive dental costs between 2010 and 2015 incurred slightly, but not significantly, lower dental costs at age 17 than those without consecutive dental costs (mean difference −13.29 euros [−28.00 to 1.42]). Adolescents with a migration background incurred higher dental costs at age 17 than those with no migration background. Regarding the level of education: those in lower vocational education, higher secondary, higher vocational, and university education incurred lower dental costs than those in lower secondary education. A higher household income was significantly associated with lower dental costs at age 17. Also, a medium or high parental educational level was associated with lower dental costs at age 17 compared to a low parental level of education. Regarding the analysis with consecutive dental costs, adolescents living with a single parent incurred lower dental costs at age 17 than their peers (Tables 2 and 3).

### Distribution of dental costs at age 9, 12, and 17

At age 9 (2009), only 14% of the children incurred no dental costs; this proportion increased steadily to 24% by age 17 (2016). The percentage of children with dental costs of up to 100 euros decreased substantially from 32% at age 9 to 19% at age 17. The largest proportion of children in Amsterdam incurred dental costs between 100 euros and 300 euros per year, although this percentage declined from 41 to 34% over the study period. In contrast, the proportion of children with high dental costs (≥ 300 euros) increased from 13% at age 9 to 23% at age 17 (Figure 3).

## Discussion

In Amsterdam, three-quarters of 17-year-old adolescents incurred dental costs in 2016, which was a proxy for visiting a dentist that year, with a mean amount of 255.63 euros per adolescent. Results showed a slight decrease in dental attendance from 85% at age 9 to 76% at 17 years of age. Children with a dental visit at age 9 were more likely to visit a dentist at age 17, and those with a higher volume of oral healthcare consumption at age 9 had even higher odds of a dental visit at age 17. Adolescents with regular dental attendance from age 10 to 16 were also more likely to visit a dentist at age 17. For 17-year-olds with a dental visit, the amount of dental costs was significantly associated with the volume of oral healthcare consumption at age 9, but there was no significant association with regular dental attendance.

Visiting a dentist at age 9 was associated with going to a dentist years later, at age 17. Also, those with a larger volume of oral healthcare consumption earlier in childhood were more likely to see a dentist when they were on the verge of adulthood. Among adolescents who incurred dental costs at age 17, every 100-euro higher dental cost at age 9 was associated with 19-euro higher dental cost at age 17 (95% confidence interval [CI]: 15 euro to 23 euro). This finding shows that higher dental costs, and thus a higher volume of oral healthcare consumption, during childhood is associated with higher dental costs at the end of adolescence. The assumption is that children with high dental costs at a young age continue to receive substantial oral healthcare, possibly due to a higher treatment need or a high caries risk. This is in line with results from previous research, where different trajectories on dental caries were shown throughout adolescence and adulthood for those with a high versus low caries risk [24, 25]. Another explanation could be variation in treatment among dentists. The threshold to provide oral healthcare may vary individually: some dentists more often apply preventive care and/or restorative treatment, compared to their colleagues, and therefore the volume of oral healthcare provided differs among dentists [26]. Because there were no data available on oral healthcare providers, this factor was not taken into account in this study.

More adolescents with regular dental attendance between the ages of 10 and 16 went to a dentist at age 17 than those without regular dental attendance. However, for 17-year-olds who visited a dentist, regular dental attendance throughout the years was not significantly associated with lower dental costs. There was only a small difference in dental costs between those with regular versus irregular dental attendance. This is in disagreement with previous literature that showed that visiting a dentist regularly for check-ups to detect oral problems in a timely manner is associated with a lower treatment need, and thus lower dental costs in the future [7, 8, 27]. In a Norwegian study, regular dental visits with 12–24-month intervals were associated with more beneficial oral health outcomes, compared to more frequent or rare, incidental dental attendance [28]. Although dental costs were similar among adolescents with and without regular dental attendance, the available data do not allow us to determine whether this reflects a similar treatment need or differences in the type or intensity of oral healthcare provided. One possible explanation is that check-ups and preventive care are provided irrespective of individual caries risk. However, because performance codes and clinical risk data were unavailable, this hypothesis could not be tested. In the short term, this approach does not reduce dental costs for those with regular dental attendance, but it is likely to yield benefits in early adulthood. However, the follow-up period of this study did not extend into adulthood [15, 29]. Another possible explanation is that socioeconomic, cultural, and family factors may have a stronger association with oral health and dental attendance, thereby limiting the influence of regular dental attendance during adolescence on dental costs at age 17 [18]. Nevertheless, frequent dental attendance seems justified for Dutch adolescents: even though oral health has improved over the last decades, caries still represents a substantial disease burden in the Netherlands [30]. In a recent national population-based study on oral health, only one-third of the 17-year-old adolescents, from both high and low SEP families, had no caries experience. For those with caries, the disease burden was substantial: mean Decayed Missing Filled Surfaces (DMFS) scores 5.6 and 3.7 for children from low and high SEP families, respectively [31].

The most important sociodemographic characteristics were migration background, household income, and parental educational level, which is in agreement with previous studies [32, 33]. Adolescents from families with a low socioeconomic position have higher odds of developing dental caries, compared to their peers [34, 35]. Early dental caries is a significant predictor of caries in adulthood and also shows a social gradient: greater caries progression is observed in adolescents with the abovementioned characteristics. Consequently, preventive care may not achieve the same (positive) effect on oral health outcomes for all groups, potentially contributing to differences in subsequent oral healthcare consumption [36, 37]. The sociodemographic characteristics of this study population of adolescents differed from the group of primary school children in Amsterdam [16], and contained a smaller proportion of adolescents with no migration background (34.7% vs. 43.7%), from a family with a high household income (13.5% vs. 21.7%) and a high parental educational level (21.8% vs. 43.4%) [16]. Those differences may be explained by the migration of young families out of the city.

The dataset analyzed in this study contained a representative sample of all 12-year-old adolescents living in Amsterdam, with valid data obtained from Statistics Netherlands, a trustworthy source. In this retrospective, longitudinal study, data were available from 2009 onward over a period of 8 years (from 9 to 17 years of age), which is a relatively short time span to analyze trends in dental costs. Therefore, the observed associations between dental costs at age 9 and dental costs at age 17 should be interpreted as associations within the available observation period, and do not reflect the full oral healthcare trajectory from early childhood onwards. Unfortunately, those data could not be analyzed by type of provided oral healthcare as no dental performance codes were available. It was assumed that low dental costs reflected a combination of routine check-ups and preventive care, whereas higher dental costs were more likely to indicate additional restorative treatment. However, this assumption could not be verified using the available data. In addition, no information was available on the adolescents’ oral health status, which meant that potential associations between oral health and patterns of oral healthcare consumption could not be explored. These assumptions limited the interpretation of oral healthcare consumption patterns. Therefore, future research should include data on the type of oral healthcare provided as well as clinical outcomes in order to align oral health policy with the patterns of oral healthcare consumption and treatment need of children in Amsterdam.

Although this study included all 12-year-old adolescents living in Amsterdam on January 1, 2012, instead of a sample, unmeasured factors may still have contributed to the observed associations. Baseline oral health status, previous caries experience, family health-seeking behavior, and variation in dentists’ treatment thresholds may have influenced dental costs at both age 9 and age 17. Since data on clinical oral health status, treatment codes, family health-seeking behavior, and dental care providers were not available, these factors could not be accounted for in the analyses. Therefore, the findings should be interpreted as associations between earlier and later reimbursed dental service use, rather than as evidence of causal effects or differences in treatment need.

Regarding oral healthcare utilization, a higher proportion of children in Amsterdam did not receive an annual dental check-up compared to the Netherlands overall (23.5% vs. 19.5%; [13]). Additionally, Amsterdam has a significantly larger population with a migration background than the national average (60% vs. 28%; [20]), as well as a relatively high proportion of families in both the highest and lowest household income groups compared to the rest of the Netherlands (17 and 14%) in Amsterdam versus the highest and lowest deciles (10%) in the Netherlands [38]. Therefore, the findings should be interpreted with caution when generalizing beyond the study population, particularly to less urban regions. The data used in this study were collected before the COVID-19 pandemic. However, there are no clear indications that oral healthcare for children and adolescents in the Netherlands changed substantially after the pandemic, and the reimbursement system for dental care for individuals under 18 years has remained largely unchanged over the last decades. Therefore, although temporal changes in attendance patterns or care provision cannot be fully excluded, the pre-COVID timing of the data is unlikely to fundamentally alter the interpretation of the findings.

In conclusion, visiting a dentist at age 9 was associated with visiting a dentist at age 17. A large volume of oral healthcare consumption at age 9 was associated with higher dental costs and a more extensive dental treatment need at age 17. Those who regularly visited the dentist throughout their adolescence continued to do so at age 17. However, regular dental attendance did not seem to be associated with lower dental costs and thus a lower dental treatment need, since there was only a small, but not significant, difference in euros between the two groups. Additional research is needed to further explore patterns of dental costs and oral healthcare consumption; specifically, why adolescents with a large volume of oral healthcare consumption during childhood continue to have high dental costs in the future, and whether different patterns of oral healthcare consumption are associated with the oral health status of adolescents.

## Data Availability

The underlying data belong to Statistics Netherlands and are not publicly available.

## References

[1] Global oral health status report – towards universal health coverage for oral health by 2030. Geneva: World Health Organization. 2022.

[2] Eurostat – statistics explained: consulting a dentist. 2021. [cited 2024 Oct 16] Available from: https://ec.europa.eu/eurostat/statistics-explained/index.php?title=Healthcare_activities_statistics_-_consultations

[3] Winkelmann J, Gomez Rossi J, van Ginneken E. Oral health care in Europe: financing, access and provision. Health Syst Transit. 2022;24(2):1–176. 10.1093/eurpub/ckac129.372 35833482

[4] Mondzorg in beeld [Report ‘Oral healthcare depicted’]. Amsterdam: ABN AMRO Bank; 2024.

[5] Winkelmann J, Gomez Rossi J, Schwendicke F, Dimova A, Atanasova E, Habicht T, et al. Exploring variation of coverage and access to dental care for adults in 11 European countries: a vignette approach. BMC Oral Health. 2022;22(1):65. 10.1186/s12903-022-02095-4 PMC8905841 35260137

[6] Richtlijn Mondzorg voor Jeugdigen preventie en behandeling van cariës [Guideline for oral healthcare for children -prevention and treatment of caries]. Utrecht: Kennisinstituut Mondzorg [Dutch Institute of Expertise for Oral Healthcare]; 2020.

[7] Thomson WM, Williams SM, Broadbent JM, Poulton R, Locker D. Long-term dental visiting patterns and adult oral health. J Dent Res. 2010;89(3):307–11. 10.1177/0022034509356779 PMC2821461 20093674

[8] Crocombe LA, Broadbent JM, Thomson WM, Brennan DS, Poulton R. Impact of dental visiting trajectory patterns on clinical oral health and oral health-related quality of life. J Public Health Dent. 2012;72(1):36–44. 10.1111/j.1752-7325.2011.00281.x 22316176

[9] Lee JJM, Schluter PJ, Hodgett M, Deng B, Hobbs M. Adolescents and oral health service utilization in Canterbury, New Zealand: a geospatial cross-sectional study. Community Dent Oral Epidemiol. 2023;51(3):388–98. 10.1111/cdoe.12858 37020331

[10] Amarasena N, Luzzi L, Brennan D. Effect of different frequencies of dental visits on dental caries and periodontal disease: a scoping review. Int J Environ Res Public Health. 2023;20(19):6858. 10.3390/ijerph20196858 PMC10572504 37835128

[11] Staat van de mondzorg – tandartsbezoek [State of the oral healthcare]. [cited 2024 Oct 16]. Available from: https://www.staatvandemondzorg.nl

[12] Gezondheidsenquête [National health survey]. CBS [Statistics Netherlands]; 2023 [cited 2024 Oct 16].

[13] Vereniging van Nederlandse Gemeenten [The Association of Netherlands Municipalities]. 2016–2021. [cited 2024 Oct 16]. Available from: https://www.waarstaatjegemeente.nl/dashboard/dashboard/gezondheid.

[14] Zorginstituut Nederland [National Health Care Institute] – tandarts en mondzorg [Dentist and oral healthcare]. [cited 2024 Oct 16]. Available from: https://www.zorginstituutnederland.nl/Verzekerde+zorg/tandarts-en-mondzorg-zvw.

[15] Staat van de mondzorg – preventieve en curatieve zorg [State of the oral healthcare] incl infographic Preventive treatment for children. 2022. [cited 2024 Oct 16]. Available from: https://www.staatvandemondzorg.nl

[16] van Ligten TS, Duijster D, Zaura E, Volgenant CMC. Influence of early and regular dental visits on dental health care costs of primary school children in Amsterdam. Int Dent J. 2025;75(4):100839. 10.1016/j.identj.2025.100839 PMC12163377 40450844

[17] van Spreuwel P, van der Heijden G, Su N, van Loveren C, Jerkovic-Cosic K. Effective Prevention of Early Childhood Caries in Well-Baby Clinics: Results of a Pragmatic Randomized Trial. Caries Res. 2025;59(6):529-43. 10.1159/000545116 PMC12060830 40101698

[18] Verlinden DA, Reijneveld SA, Lanting CI, van Wouwe JP, Schuller AA. Socio-economic inequality in oral health in childhood to young adulthood, despite full dental coverage. Eur J Oral Sci. 2019;127(3):248–53. 10.1111/eos.12609 PMC6593773 30791128

[19] Royal Dutch Dental Association. Tarievenlijst tandheelkundige zorg [List of Dutch dental performance codes]. 2016.

[20] Migratieachtergrond [Migration background]. The Hague: CBS [Statistics Netherlands]; 2021.

[21] Rijksoverheid – voortgezet onderwijs [Government of the Netherlands- secondary education]. Available from: https://www.rijksoverheid.nl/onderwerpen/voortgezet-onderwijs

[22] Opleidingsniveau [Educational level]. The Hague: CBS [Statistics Netherlands]; 2020.

[23] IBM Corp. IBM SPSS statistics for windows, Version 25.0. Armonk, NY: IBM Corp; 2017.

[24] Broadbent JM, Foster Page LA, Thomson WM, Poulton R. Permanent dentition caries through the first half of life. Br Dent J. 2013;215(7):E12. 10.1038/sj.bdj.2013.991 24113990

[25] Nowak AJ, Casamassimo PS, Scott J, Moulton R. Do early dental visits reduce treatment and treatment costs for children? J Mich Dent Assoc. 2016;98(1):36–42. 26882647

[26] Baadoudi F, Maskrey N, Listl S, van der Heijden GJ, Duijster D. Improving oral healthcare: towards measurement? Br Dent J. 2016;221(9):547–8. 10.1038/sj.bdj.2016.813 27811893

[27] Brennan DS, Spencer AJ. Influence of patient, visit, and oral health factors on dental service provision. J Public Health Dent. 2002;62(3): 148–57. 10.1111/j.1752-7325.2002.tb03436.x 12180042

[28] Nermo H, Hadler-Olsen E. Are dental visiting patterns and oral pain associated with dental disease among Norwegian adults? A cross-sectional study based on the Tromso study. Clin Exp Dent Res. 2023;9(4):679–88. 10.1002/cre2.753 PMC10441595 37415297

[29] Ghanem AS, More M, Nagy AC. Assessing the impact of sociodemographic and lifestyle factors on oral health: a cross-sectional study in the Hungarian population. Front Public Health. 2023;11:1276758. 10.3389/fpubh.2023.1276758 PMC10623121 37927886

[30] Signalement Mondzorg [Description of oral healthcare]. Diemen: Zorginstituut Nederland [National Health Care Institute]; 2018.

[31] Schuller AA, Vermaire JH, Verrips GHW. [Kies-voor-Tanden Study: caries among 17-year-olds in the Netherlands]. Ned Tijdschr Tandheelkd. 2020;127(2):109–17. 10.5177/ntvt.2020.02.19088 32271328

[32] Julihn A, Ekbom A, Modeer T. Migration background: a risk factor for caries development during adolescence. Eur J Oral Sci. 2010;118(6): 618–25. 10.1111/j.1600-0722.2010.00774.x 21083624

[33] Bissar AR, Oikonomou C, Koch MJ, Schulte AG. Dental health, received care, and treatment needs in 11- to 13-year-old children with immigrant background in Heidelberg, Germany. Int J Paediatr Dent. 2007;17(5):364–70. 10.1111/j.1365-263X.2007.00846.x 17683326

[34] Brennan DS, Spencer AJ. Childhood oral health and SES predictors of caries in 30-year-olds. Caries Res. 2014;48(3):237–43. 10.1159/000354044 24481125

[35] Singh A, Peres MA, Watt RG. The relationship between income and oral health: a critical review. J Dent Res. 2019;98(8):853–60. 10.1177/0022034519849557 31091113

[36] Kallestal C, Wall S. Socio-economic effect on caries. Incidence data among Swedish 12–14-year-olds. Community Dent Oral Epidemiol. 2002;30(2):108–14. 10.1034/j.1600-0528.2002.300204.x 12000351

[37] Hall-Scullin E, Whitehead H, Milsom K, Tickle M, Su TL, Walsh T. Longitudinal study of caries development from childhood to adolescence. J Dent Res. 2017;96(7):762–7. 10.1177/0022034517696457 28571506

[38] Huishoudinkomen [Household income]. The Hague: CBS [Statistics Netherlands]; 2025.

